# miR-182 modulates cell proliferation and invasion in prostate cancer via targeting ST6GALNAC5

**DOI:** 10.1590/1414-431X2020e9695

**Published:** 2021-05-24

**Authors:** Liang Bai, Li Luo, Weicheng Gao, Chenfeng Bu, Jianfeng Huang

**Affiliations:** 1 Department of Urology, The First Affiliated Hospital, School of Clinical Medicine of Guangdong Pharmaceutical University, Guangzhou, China; 2 Department of Urology, People's Hospital of Liannan Yao Autonomous County, Qingyuan, China

**Keywords:** miR-182, ST6GALNAC5, Prostate cancer, Proliferation, Invasion

## Abstract

Altered expression of miR-182 has been observed in various types of human cancer. The purpose of this study was to investigate the expression of miR-182 and its role in prostate cancer (PCa). Expression of miR-182 and ST6GALNAC5 in tumor tissues and the Du145 PCa cell line was analyzed. Cell proliferation assay, colony formation assay, transwell assay, and wound healing assay were performed. The impact of miR-182 on tumor growth was investigated using a xenograft model. The results indicated that expression of miR-182 was higher in PCa tissues and cell lines, while ST6GALNAC5 was decreased. Downregulating miR-182 significantly inhibited the capacities of proliferation and invasion of PC3 and Du145 cells. ST6GALNAC5 was demonstrated to be a target of miR-182 by luciferase assay, and western blot results indicated PI3K/Akt pathway was involved in miR-182 associated effects on PC3 and Du145 cells. The animal experiment suggested that knockdown of miR-182 inhibited tumor growth. Our study proved that miR-182 participated in the proliferation and invasion of PCa cells via mediating expression of ST6GALNAC5 and established a miR-182/ST6GALNAC5/PI3K/AKT axis in regulation of tumor progression. Our investigation provided a basis for further exploration of the application of miR-182 or ST6GALNAC5-associated therapies for PCa patients.

## Introduction

Human prostate cancer (PCa) is one of the most common malignant tumors, especially for aging males, and frequently known as the fourth most prevalent cause of cancer-related mortality worldwide (1,2). Despite major improvements of diagnosis and therapy strategies, the clinical benefits are still unsatisfactory (2). Most patients experience recurrence and poor prognosis after the standard treatments (2). Studies have revealed that the high recurrence rate is mainly due to invasion, migration, and proliferation capacities of PCa cells (1,2). Aberrant alterations at the protein and gene levels have been recognized as one of the reasons for high invasion, migration, and proliferation of tumor cells, however, the inherent molecular underpinnings are still unclear (3–7). Thus, the mechanisms underlying the poor prognosis as well as novel therapeutic strategies of PCa must be explored.

MicroRNAs (miRNAs) are a cluster of endogenous non-coding RNAs with 20-22 nucleotides (2). miRNAs can negatively regulate gene expression by repressing the translation of target mRNAs and having important roles in some essential biological processes including cellular proliferation, differentiation, apoptosis, and tumorigenesis (3,4,7). An increasing body of investigation has identified the aberrant expression of miRNAs in multiply cancers including breast cancer (3,4), lung cancer (5–7), colorectal cancer (8), pancreatic cancer (9), and PCa (10,11). Emerging studies have established a critical role of miR-182 in regulation of tumor progression. For example, the increased expression of miR-182 promoted cell proliferation and invasion by regulating NDRG1 (1), GNA13 (12), and BRCA1expression (13), among others, in prostate cancer. It was also demonstrated that miR-182 is involved in chemoresistance of non-small cell lung cancer via downregulating PDCD4 (5). The diverse effects of miR-182 on tumor progression were presumably attributed to the various downstream targeting genes and the roles of miR-182 in PCa deserve more attention. *ST6GALNAC5* gene encodes an α2,6-sialyltransferase mediating sialylation (14). A review summarized that expression of sialylation was abnormal in most malignancies with high invasiveness and metastatic potential (15). ST6GALNAC5 has been reported to be involved in metastases of breast cancer (14,16,17). ST6GALNAC2, another sialyltransferase, was proven to be targeted by miR-182 and involved in migration, adhesion, invasion, and proliferation in colorectal cancer cells. Bioinformatics analysis in this study indicated *ST6GALNAC5* as a target gene of miR-182, while investigations towards the effects of ST6GALNAC5 on PCa are lacking. Therefore, the roles of miR-182 and ST6GALNAC5 in PCa progression were explored here.

## Material and Methods

### Tissue samples

A total of 25 tumor tissue samples (median Gleason score of seven) and paired adjacent noncancerous tissue samples that were 2 cm away from the lesion were collected from patients receiving surgical resection at the Department of Urology, The First Affiliated Hospital/School of Clinical Medicine of Guangdong Pharmaceutical University between May 2012 and Dec 2018. All PCa patients were histologically diagnosed by staining with hematoxylin and eosin (H&E), and the adjacent noncancerous tissues were also confirmed by H&E staining. Clinicopathological characteristics of all patients were analyzed by a pathologist. None of the patients received any anti-tumor treatment prior to surgery. Written informed consent was obtained from all enrolled patients, and the study was approved by the ethics committee of The First Affiliated Hospital.

### Cell culture

Human PCa cell lines (Du145, PC3, LNCap), normal human prostate epithelial (NHPE) cells, and HEK 293T were purchased from American Type Culture Collection (ATCC). Du145, PC3, LNCap were respectively maintained in minimum Eagle's medium, F-12K, and RPMI 1640 (GIBCO, USA) as recommended by ATCC, while HEK 293T and NHPE were maintained in Dulbecco's Modified Eagle's medium (GIBCO). All media used were supplemented with 10% fetal bovine serum (GIBCO) in a humidified incubator at 37°C under 5% CO_2_.

### RNA extraction and qRT-PCR assay

Total RNA was extracted using RNeasy Plus Micro Kit (QIAGEN, USA). Quality and concentration were determined by an ultraviolet spectrophotometer (Thermo, USA). cDNA was prepared from 1000 ng of RNA using Prime Script™ RT-PCR kit (Takara, Japan). All primers were designed on the website of National Center for Biotechnology Information (https://www.ncbi.nlm.nih.gov/tools/primer-blast/). cDNA for miR-182 detection was synthesized using M-MLV MicroRNA Reverse Transcription Kit (Promega, USA). Reverse transcription primer for miR-182 was 5′-GTCGTATCCAGTGCAGGGTCCGAGGTGCACTGGATACGACAGTGTGA-3′ and for U6 was 5′-GTCGTATCCAGTGCAGGGTCCGAGGTGCACTGGATACGACAAAATATGG-3′. The sequences of primers for quantitative real-time PCR (qRT-PCR) are listed in Table 1, qRT-PCR was performed in triplicate using SYBR green mixture (CWBIO, China), and the qRT-PCR program consisted of initial denaturing at 95°C for 10 min, followed by 40 cycles of 10 s at 95°C and 60 s at 60°C. Relative expression levels of miR-182 and ST6GALNAC5 were calculated using 2^-ΔΔCt^ rate method. MiR-182 expression was normalized to U6, while the expression level of ST6GALNAC5 was normalized to GAPDH. NHPE was used as normalizer in cell samples. The sample that was detected with the maximum Ct value was used as normalizer in tissue samples.

**Table 1:** Sequence of primers.

Primer	Sequence 5′-3′
GAPDH	Forward GGTGAAGGTCGGAGTCAACG
	Reverse ACCATGTAGTTGAGGTCAATGAAGG
ST6GALNAC5	Forward GGATCCCAATCACCCTTCAG
	Reverse TAGCAAGTGATTCTGGTTTCCA
miR-182	Forward TGCGGTTTGGCAATGGTAGAAC
	Reverse CCAGTGCAGGGTCCGAGGT
U6	Forward TGCGGGTGCTCGCTTCGGCAGC
	Reverse CCAGTGCAGGGTCCGAGGT

### Western blot analysis

Protein was extracted using RIPA lysis buffer (Cell Signaling Technology, USA) supplemented with protease inhibitor and phosphatase inhibitor. Concentration was determined by the Pierce Coomassie (Bradford) Protein Assay Kit (Thermo). Then, samples were developed on 8% SDS-PAGE at 90 volts and transferred to polyvinylidene difluoride membranes at 250 mA electric current. The membranes were blocked with 5% fat-free milk for 1 h at room temperature and incubated with the primary antibodies targeting human GAPDH (Cell Signaling Technology, Cat 8884), PI3K (Cell Signaling Technology, Cat 3011), P-Akt (Cell Signaling Technology, Cat 4060), Akt (Cell Signaling Technology, Cat 02920), or ST6GALNAC5 (Abcam, UK, Cat ab201575) overnight at 4°C. After washing, the membrane was incubated with anti-rabbit (Cell Signaling Technology, Cat 7074) or anti-mouse (Cell Signaling Technology, Cat 58802) IgG secondary antibody conjugated with horseradish peroxidase. The signal was developed using Super Signal West Dura Extended Duration chemiluminescence substrate (Thermo) and measured by the ChemiDoc™ XRS+ system (Bio-Rad, USA).

### Construction of vectors and stable transduction of PCa cells

Short hairpin RNA (shRNA) targeting ST6GALNAC5 (sh-ST6) or miR-182 (sh-miR-182) were designed and constructed by GenePharma Co., Ltd. (China). Sh-ST6 or sh-miR-182 was subcloned into the lentiviral vector pLKO.1-TRC cloning vector (Sigma, USA). A PLKO.1-scramble vector with limited homology with any human gene sequences was used as the negative control (NC). PCa cells were transduced with the shRNA vector or NC. Puromycin (2 μg/mL, Sigma) was used after transduction for 48 h to select the stably transduced cells.

### Proliferation assay

For the cell proliferation assay, PC3 and Du145 cells transduced with the indicated shRNAs or NC lentivirus were plated onto a flat bottom 96-well plate at 3×10^3^ cells per well in triplicate with 100 µL medium. A 10-µL volume of cell counting kit-8 (CCK-8, Dojindo, China) reagent was added to each well. After incubation for 2 h, absorbance at 450_nm_ was measured using a microplate reader (Bio-Rad). Absorbance values were detected after cell culturing for 24, 48, 72, 96, and 120 h.

### Colony formation assay

PC3 and Du145 cells transduced with shRNAs or NC lentivirus were plated in 6-well plates and cultured with 2 mL of indicated medium for two weeks. Then, the cells were fixed with 4% paraformaldehyde for 1 h and stained with 0.1% crystal violet overnight. After washing with PBS three times, cell colonies were photographed and counted.

### Transwell assay

A total of 4×10^4^ PC3 or Du145 cells in 100 μL serum-free medium were plated into the upper chamber of an 8-μm-pore membrane (Costar, USA) coated with Matrigel (BD, USA) and 600 μL complete medium was added as a chemoattractant to the lower chamber. After 36 h incubation, the non-invading cells were scraped with a cotton swab and the membranes were then fixed with 100% methanol and stained with 0.5% crystal violet. Membranes were photographed using a digital light microscope and five fields were selected randomly to obtain the mean cell number on each membrane.

### Luciferase assay

The miR-182 binding sites in wild-type (WT) 3′-untranslated regions (3′-UTR) of *ST6GALNAC5* was amplified and cloned downstream of the firefly luciferase gene in the pGL3-control vector (Promega). Subsequently the mutant (Mut) 3′-UTR of *ST6GALNAC5* was created by site-directed mutagenesis. Mimics (miR-182-mimic) and negative control (NC) oligonucleotides for miR-182 were purchased from RiboBio Co., Ltd. (China). HEK 293T cells (5×10^4^) were seeded onto a 24-well plate for 24 h. miR-182-mimic or NC together with pGL3-control vector carrying wild-type or mutant 3′-UTR of *ST6GALNAC5* were co-transfected into HEK 293T cells using Lipofectamine 2000 reagent (Promega, USA) following instructions. After incubation for 48 h, the activities of firefly and renilla luciferases were measured using the Dual Luciferase Assay Kit (Promega) and normalized to those of firefly luciferase activity. All assays were designed in triplicate and repeated for six times.

### Animal experiments

Twelve NOD/SCID mice (female, 6-8 weeks) purchased from SLAC Laboratory Animal Co., Ltd. (China) were equally and randomly divided into two groups. Ten million PC3 cells transduced with NC or sh-miR-182 were subcutaneously injected into the left flank of mice. After 7 days, the tumor volume was monitored every 3 days using a caliper. After 25 days, the mice were sacrificed and the tumors were excised. Tumor volume was calculated using the formula: volume = 0.5 × length × width^2^. All animal experiments were approved by the Animal Care and Use Committee of The First Affiliated Hospital/School of Clinical Medicine of Guangdong Pharmaceutical University.

### Statistical analysis

All statistical analyses were performed using SPSS 19.0 (IBM, USA). Data are reported as means±SD of parallel experiments. The Shapiro-Wilk test was used to assess data normality and the paired *t*-test was performed to compare differences between two groups. For more than two groups, one-way ANOVA was used to compare the differences. Pearson analysis was used to determine the correlation between miR-182 and ST6GALNAC5. P<0.05 was considered as a statistically significant difference.

## Results

### Expression pattern of miR-182 and ST6GALNAC5 in PCa cell lines and tissues

**Figure 1:**
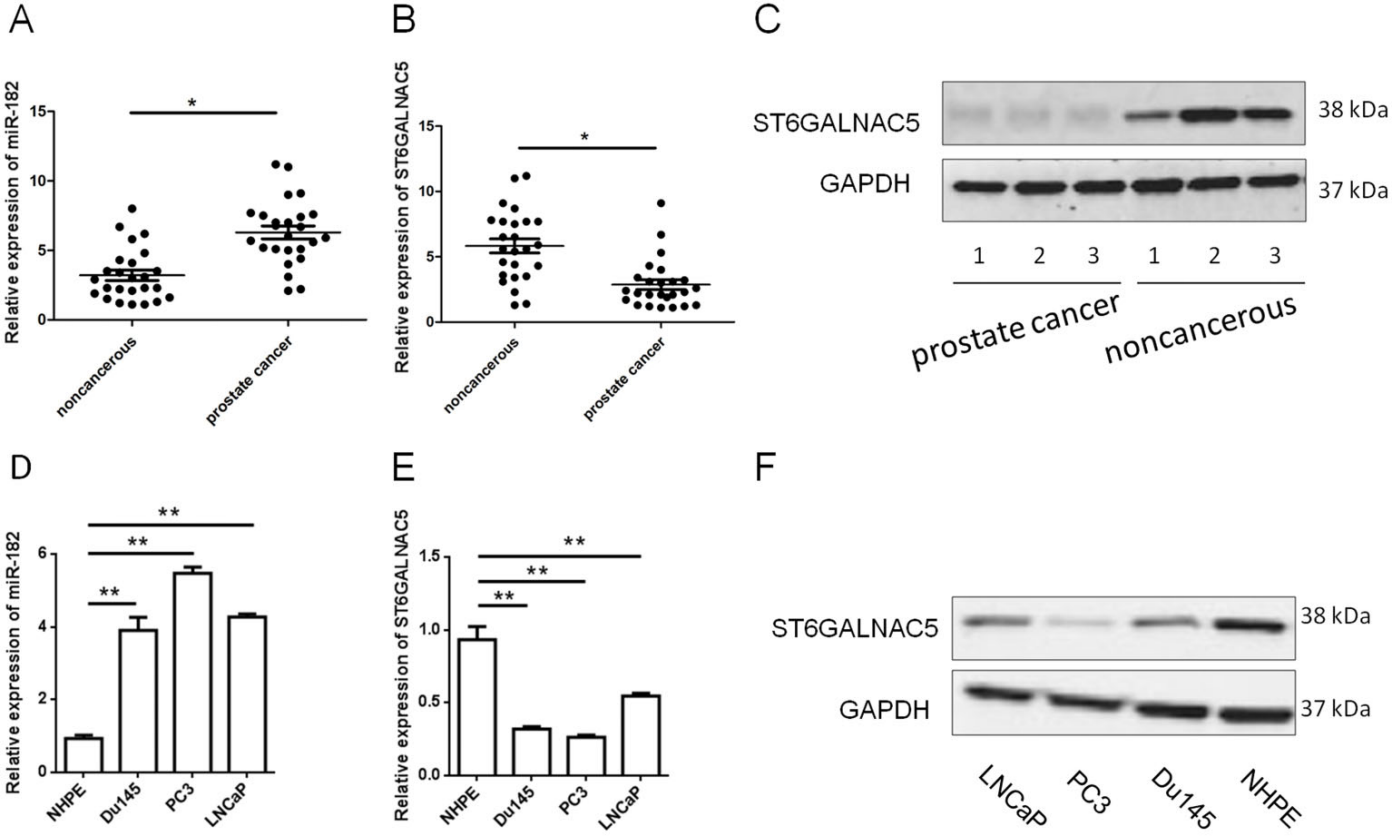
A, Expression of miR-182 in 25 pairs of prostate cancer (PCa) tissue and adjacent noncancerous tissue samples were analyzed by qRT-PCR. **B** and **C**, ST6GALNAC5 in tissues was also determined by qRT-PCR and western blot. **D**, Expressions of miR-182 and (**E** and **F**) ST6GALNAC5 in PCa cell lines were analyzed by qRT-PCR. Data are reported as means±SD. *P<0.05; **P<0.01 (paired *t*-test and one-way ANOVA).

miR-182 was significantly higher in PCa tissues compared with adjacent noncancerous tissues (Figure 1A). The data also indicated that ST6GALNAC5 was downregulated in PCa tissues (Figure 1B and C). PCa cell lines exhibited a statistically higher level of miR-182 than NHPE (Figure 1D), which was in accordance with what we found in PCa and paired noncancerous tissues. As shown in Figure 1E and F, PCa cell lines were also detected with a reduced expression of ST6GALNAC5. Association of miR-182 with clinicopathological features of PCa patients were analyzed using the chi-squared test, and results (Table 2) suggested that miR-182 expression was significantly correlated with distant metastasis (P=0.027), lymph node metastasis (P=0.028), and tumor size (P=0.025). ST6GALNAC5 expression was significantly correlated with distant metastasis (P=0.028) and tumor size (P=0.032).

**Table 2:** Correlation between the expression of miR-182, ST6GALNAC5, and clinicopathological factors of patients with prostate cancer (n=25).

Variable	miR-182 expression		P value	ST6GALNAC5		P value
	Low (n=13)	High (n=12)		Low (n=14)	High (n=11)	
Age (years)						
<60	5	5	0.870	8	6	0.897
≥60	8	7		6	5	
Lymph node metastasis						
Not detected	10	4	0.028*	5	8	0.066
Detected	3	8		9	3	
Tumor Size (cm)						
≤4.0	11	5	0.025*	3	7	0.032*
>4.0	2	7		11	4	
Clinical Stage						
I-II	8	3	0.066	5	6	0.346
III-IV	5	9		9	5	
Distant metastasis						
Not detected	9	3	0.027*	4	8	0.028*
Detected	4	9		10	3	

Low/high cutoff by the sample mean. *P<0.05 was considered statistically significant (Pearson chi-squared test).

### miR-182 silencing inhibited proliferation and invasion of PC3 and Du145 *in vitro*

**Figure 2:**
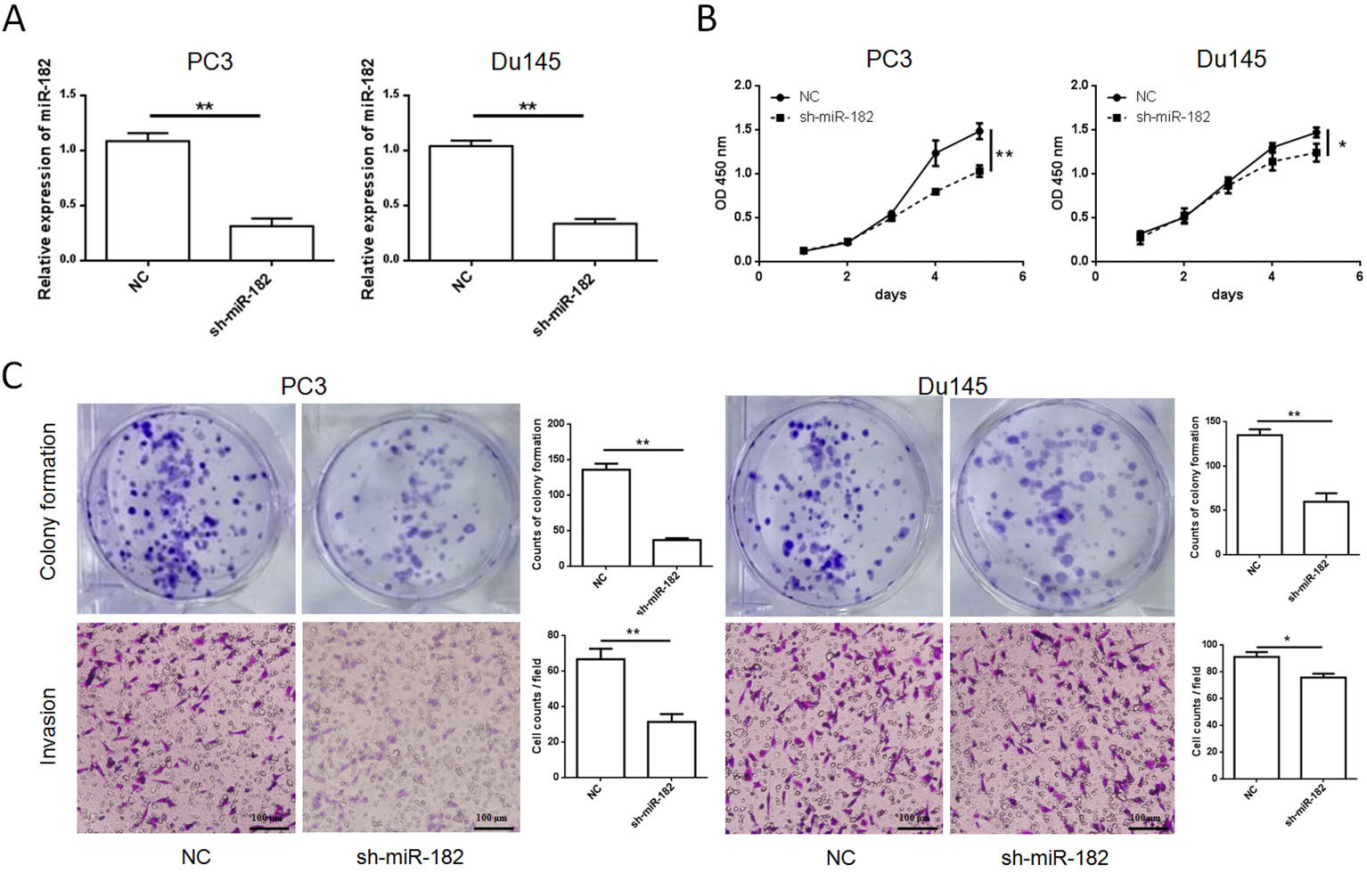
Knockdown of miR-182 inhibited prostate cancer cell proliferation and invasion *in vitro*. **A**, shRNA was designed to knockdown endogenous miR-182, and qRT-PCR indicated its expression was decreased by transduction of sh-miR-182 lentivirus in PC3 and Du145. **B**, CCK-8 assay was used to evaluate cell proliferation. **C**, Knockdown of miR-182 significantly inhibited colony formation and invasion of PC3 cells (scale bar 100 μm). Data are reported as means±SD. *P<0.05; **P<0.01 (paired *t*-test and one-way ANOVA). NC: negative control.

The knockdown effect of miR-182 was confirmed by qRT-PCR assay (Figure 2A). CCK-8 assay results suggested that a decreasing miR-182 level significantly lowered the absorbance value in the sh-miR-182 group, which indicated hindered proliferation (Figure 2B). A similar role was also validated in the same cell lines by colony formation assay where fewer counts of colony formation were observed in cells transduced with sh-miR-182 (Figure 2C). The obtained results implied that a high level of miR-182 was important for proliferation of PC3 and Du145 cells *in vitro*. Results of transwell assays illustrated that the invasive capacities of PC3 and Du145 were significantly reduced by miR-182 knockdown (Figure 2C). Taken together, these results suggested that miR-182 was essential for PCa cell invasion.

### miR-182 directly targeted ST6GALNAC5 in PC3

**Figure 3:**
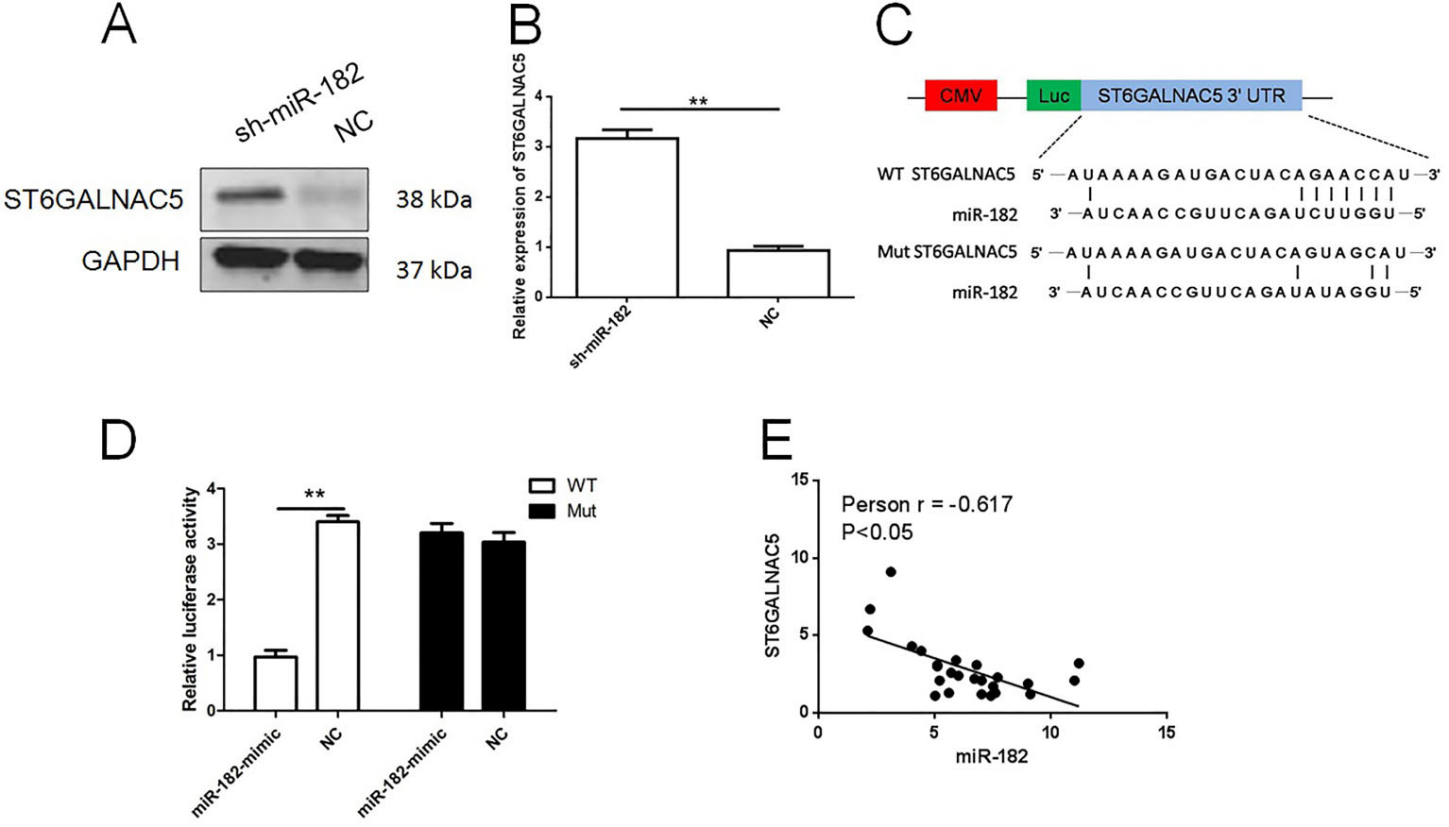
Aand **B**, Western blot and qRT-PCR assays were performed to determine ST6GALNAC5 expression in PC3 cells. **C**, Bioinformatics analysis revealed that ST6GALNAC5 was a direct target of miR-182. **D**, Luciferase reporter assay was performed in HEK 293T cells. Data are reported as means±SD. **P<0.001 (paired *t*-test). **E**, Pearson analysis indicated miR-182 level was negatively correlated with ST6GALNAC5 in prostate cancer tissues. WT: wild type; Mut: mutant; NC: negative control.

Western blot and qRT-PCR assays proved that lowering miR-182 level by stable transduction of sh-miR-182 lentivirus in PC3 significantly increased the expression of ST6GALNAC5 (Figure 3A and B). Therefore, it was speculated that miR-182 might directly modulate ST6GALNAC5 expression. Results of bioinformatics analysis using miRanda, TargetScan, and PicTar showed that the 3′-UTR of *ST6GALNAC5* contained a potential binding site for miR-182 (Figure 3C). To dissect the direct regulation of ST6GALNAC5 by miR-182, a luciferase reporter assay was performed. Co-transfection of miR-182-mimic and vectors carrying WT 3′-UTR of *ST6GALNAC5* evidently attenuated the relative luciferase activity, which was detected with no significant difference in groups transfected with Mut 3′-UTR of *ST6GALNAC5* and NC for miR-182 (Figure 3D). Also, Pearson analysis indicated that ST6GALNAC5 expression in PCa specimens was negatively correlated with miR-182 expression (Figure 3E).

### Knockdown of ST6GALNAC5 counteracted tumor-inhibiting effects caused by lowering miR-182 level in PCa cells

**Figure 4:**
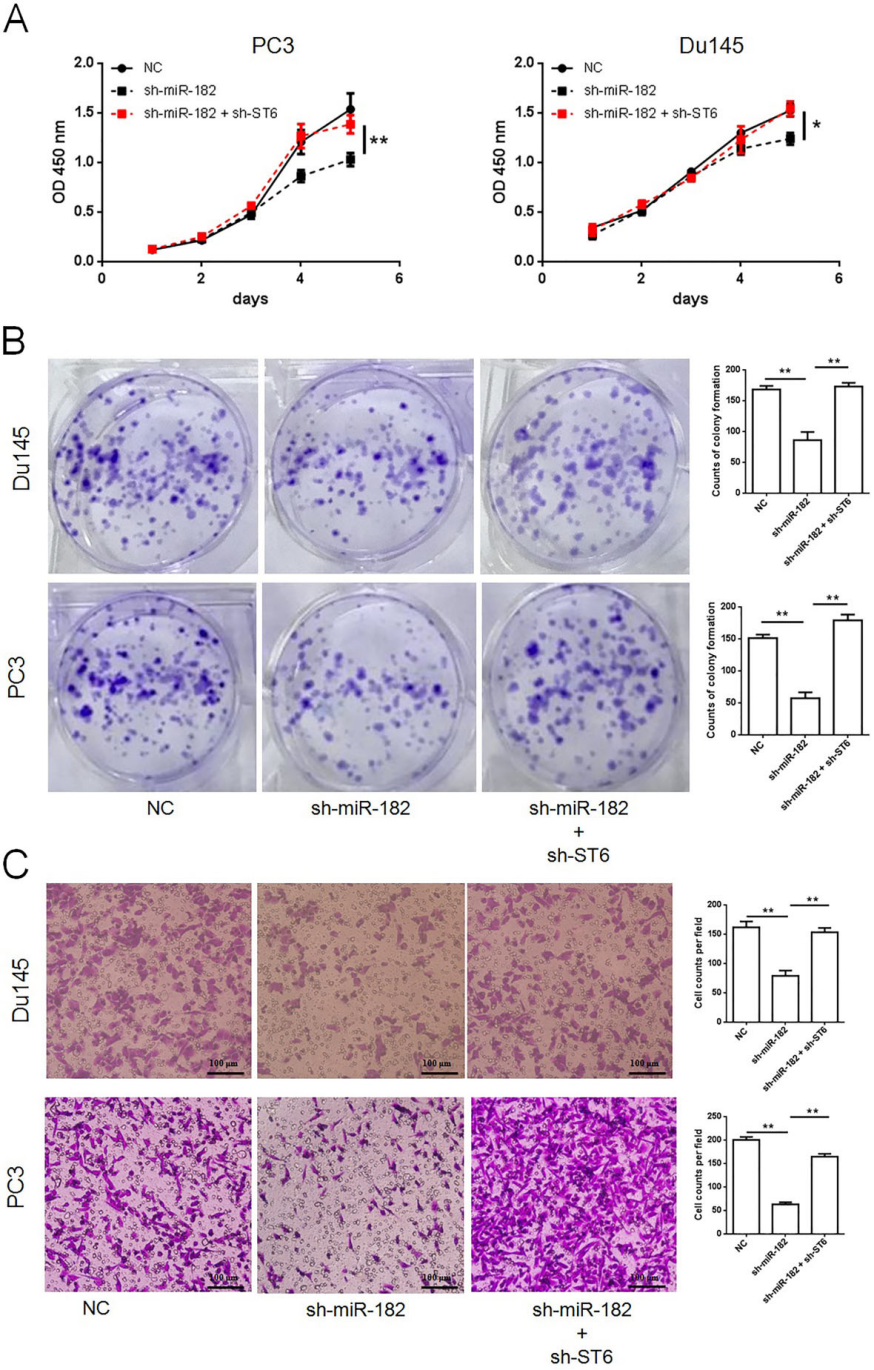
A, shRNA against ST6GALNAC5 or miR-182 was designed, and silence of both miR-182 and ST6GALNAC5 rescued cell proliferation, which was attenuated by miR-182 knockdown in PC3 and Du145. Knockdown of ST6GALNAC5 counteracted inhibition effects caused by miR-182 knockdown on colony formation (**B**) and invasion (**C**) capacities (scale bar 100 μm). Data are reported as means±SD. *P<0.05; **P<0.01 (one-way ANOVA). NC: negative control.

**Figure 5:**
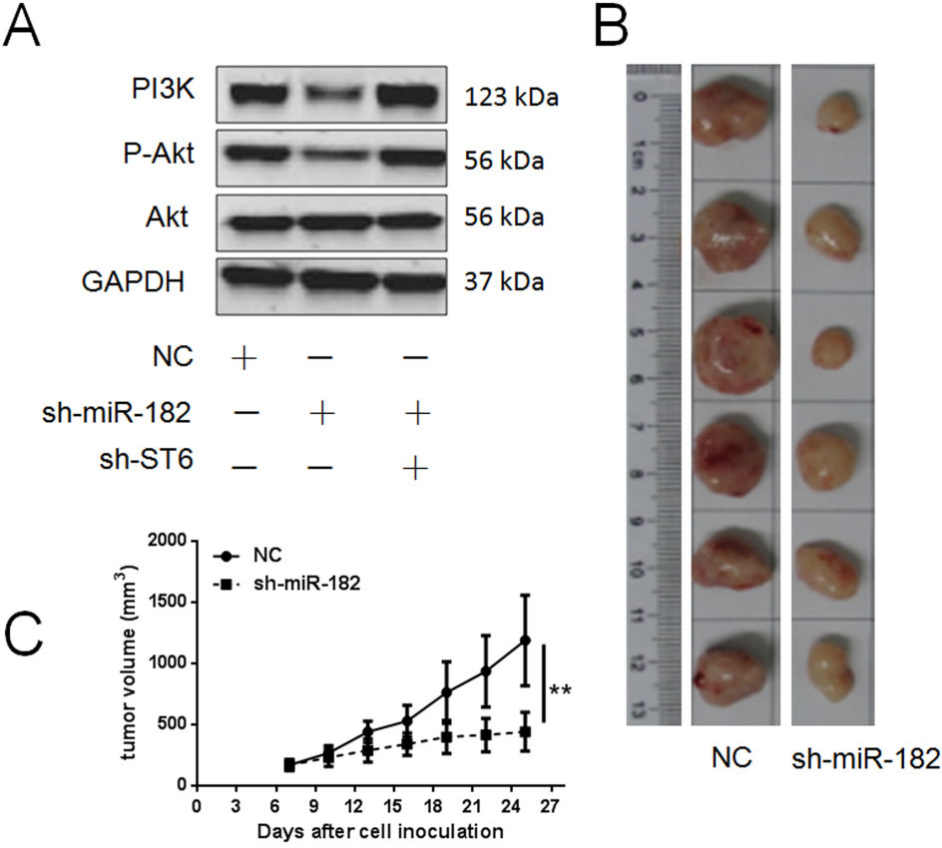
Knockdown of miR-182 inhibited tumorigenesis of PC3 cells. **A**, Western blot assay was used to detect key factors in PI3K/Akt pathway. **B**, Image of tumor formation in the negative control (NC) group and the sh-miR-182group in NOD/SCID mice. **C**, The average tumor volume in the sh-miR-182 group was smaller than in the NC group. Data are reported as means±SD. **P<0.01 (one-way ANOVA).

It was demonstrated that ST6GALNAC5 expression was directly regulated by miR-182. The downregulation of ST6GALNAC5 in PCa cell lines was probably responsible for miR-182-induced cell proliferation and invasion. shRNA targeting miR-182 (sh-miR-182) or ST6GALNAC5 (sh-ST6) was constructed to determine the mechanism mediating miR-182-induced cell proliferation and invasion. Proliferation assays suggested that the proliferation-inhibiting effect of sh-miR-182 was rescued by transduction of sh-ST6 in the same cells *in vitro* (Figure 4A), a similar result was obtained in the colony formation assay (Figure 4B). Additionally, transwell assays proved that the inhibitory effect on cell invasion caused by miR-182 knockdown was counteracted by simultaneous transduction of sh-ST6 in PCa cells (Figure 4C). The above findings revealed that the effects of miR-182 on cell proliferation and invasion was potentially achieved by its regulation of ST6GALNAC5 expression. Western blot analysis indicated that PI3K/Akt signal pathway was partially blocked when the miR-182 level was reduced; otherwise, the signal pathway was reactivated by additional decrease in ST6GALNAC5 expression (Figure 5A). These results implied that miR-182 regulated PI3K/Akt signal pathway via ST6GALNAC5 and indicated a miR-182/ST6GALNAC5/PI3K/Akt axis in PC3.

### Knockdown of miR-182 suppressed tumorigenesis in a xenograft model

PC3 cells transduced with NC shRNA or shRNA targeting miR-182 (sh-miR-182) were subcutaneously injected into NOD/SCID mice. It was observed that tumor growth was significantly slowed in the sh-miR-182 group compared to the NC group (Figure 5B and C), indicating that silencing miR-182 significantly inhibited the tumorigenesis of PC3.

## Discussion

miRNAs are a novel class of gene regulators that are involved in different malignancies (18–21). Recent research documents that miRNAs participate in diverse biological processes and serve as biomarkers in PCa (22–25). miR-182 was reported to serve as a tumor-associated miRNA in colorectal cancer and PCa (10,26). A meta-analysis on the prognostic value of miR-182 in cancers indicates that high miR-182 expression is significantly associated with poor overall survival (27). Our results also demonstrated that high miR-182 expression was correlated with advanced clinicopathological features.

In the present study, we demonstrated that the expression of miR-182 was significantly higher in PCa tissues and cell lines than that in adjacent noncancerous tissues and normal cells, which was in line with the results of a published investigation (26). Subsequently, the effects of miR-182 on PCa cell proliferation and invasion were evaluated in PCa cells. Results of CCK-8 and colony formation assays indicated that knockdown of miR-182 significantly reduced the proliferation capacity of PCa cells, and tumorigenesis was also inhibited when miR-182 was downregulated. The promotion effect of miR-182 on tumor cells was previously verified in breast cancer, ovarian cancer, pancreatic cancer, and colorectal cancer in several studies (3,4,8,9,28,29). The inherent molecular mechanism of miR-182-induced proliferation was briefly explored previously. Liu et al. proved that ectopic overexpression of miR-182 significantly promoted the G1/S cell cycle transition and reduced early apoptosis of PC3 cells, which in turn increased cell survival (1). Transwell assays demonstrated that invasion of PCa cells was decreased by reducing miR-182. Accordingly, we concluded that miR-182 was associated with PCa progression. Additionally, Yang et al. (30) found that the invasive and migratory abilities of lung cancer cells were enhanced by silencing miR-182, however, proliferation was inhibited in these cells. These results in different tumors uncovered a more complicated role of miR-182 in cancers.

Our data revealed that ST6GALNAC5 was downregulated in PCa tissues and cell lines, which was inversed with miR-182 expression. Bioinformatics analysis indicated that *ST6GALNAC5* was a direct target gene of miR-182, which was further confirmed by luciferase assays. We further demonstrated that the reduced capacities of proliferation and invasion from miR-182 silencing alone was reversed by silencing of both miR-182 and ST6GALNAC5. Previous studies reported that ST6GALNAC2, encoding another sialyltransferase, was downregulated by miR-182 and associated with tumorigenesis and invasiveness in colorectal cancer cells. The author further found that the PI3K/Akt pathway was blocked by overexpression of ST6GALNAC (8). Our data also showed that PI3K and P-Akt levels in PC3 cells were decreased when miR-182 was downregulated and the decreased PI3K and P-Akt were rescued by further reducing ST6GALNAC5 expression. These results indicated that overexpressed miR-182 decreased the expression of ST6GALNAC5 by directly binding to its 3′-UTR region. Reducing ST6GALNAC5 expression subsequently provoked the activity of PI3K/Akt pathway, finally enhancing cell proliferation and invasion. Moreover, a study focusing on colorectal cancer indicated that miR-182 might activate PI3K/Akt pathway by attenuating ST6GALNAC2 expression (8). Another investigation about prostate cancer proved that miR-182 has a tumor-promoting role via activating Wnt/β-catenin signal pathway (26). Since sialic acid plays vital roles in cell-cell communication, cell-matrix interaction, and adhesion (15), it was hypothesized that miR-182 might modulate sialylation via ST6GALNAC5, thus influencing cellular sialic acid in tumor cells. This may be a potential mechanism mediating miR-182-associated effects on PCa, which deserves more study.

Generally, one miRNA has more than one target gene. Numerous studies have proven that miR-182 has diverse target genes including *NDRG1* (1), *GNA13* (12), *MIM* (3), *PDCD4* (5), *BRCA1* (13), *SMAD7* (29), and *FOXO1* (31). Tumor angiogenesis is essential for progression of solid tumors, and miR-182 was recently reported to facilitate angiogenesis by targeting Kruppel-like factor 2/4 in glioblastoma (32). In addition, the vital roles of miR-182 are not limited to functions of tumor cells. Zhao et al. (33) showed that miR-182 promotes the polarization of macrophages from M1 to M2 by targeting Toll-like receptor 4, and an investigation has already demonstrated that M2 macrophages indicate poor prognosis in PCa (34). Different target genes of miR-182 diversify the mechanisms by which miR-182 participates in tumor progression including PCa. The present study increased our knowledge about effects of miR-182 on PCa progression. More efforts should be made to completely outline the mechanism of miR-182-induced tumor progression.

## Conclusion

This study revealed that miR-182 participated in the proliferation and invasion of PCa cells and demonstrated that ST6GALNAC5 was partially responsible for the miR-182-induced cell proliferation and invasion. The findings of our study provide the basis for further exploration of the application of miR-182 as a prognostic and diagnostic biomarker.
